# Exploring the Adhesion Properties of Extracellular Vesicles for Functional Assays

**DOI:** 10.1002/jex2.70042

**Published:** 2025-04-25

**Authors:** Bianca C. Pachane, Bess Carlson, Suzanne E. Queen, Heloisa S. Selistre‐de‐Araujo, Kenneth W. Witwer

**Affiliations:** ^1^ Department of Molecular and Comparative Pathobiology Johns Hopkins University School of Medicine Baltimore Maryland USA; ^2^ Biochemistry and Molecular Biology Laboratory, Department of Physiological Sciences Universidade Federal de São Carlos – UFSCar São Carlos São Paulo Brazil

**Keywords:** adhesion, extracellular vesicles, super‐resolution microscopy

## Abstract

The ‛stickiness’ of extracellular vesicles (EVs) can pose challenges for EV processing and storage, but adhesive properties may also be exploited to immobilise EVs directly on surfaces for various measurement techniques, including super‐resolution microscopy (SRM). Direct adhesion to surfaces may allow the examination of broader populations of EVs than molecular affinity approaches, which can also involve specialised, expensive affinity reagents. Here, we report on the interaction of EVs with borosilicate glass and quartz coverslips and on the effects of pre‐coating coverslips with poly‐L‐lysine (PLL), a reagent commonly used to facilitate interactions between negatively charged surfaces of cells and amorphous surfaces. Additionally, we compared two mounting media conditions for SRM imaging and used immobilised EVs for a B‐cell interaction test. Our findings suggest that borosilicate glass coverslips immobilise EVs better than quartz glass coverslips. We also found that PLL is not strictly required for EV retention but contributes to the uniform distribution of EVs on borosilicate glass coverslips. Overall, these findings suggest that standard lab materials like borosilicate glass coverslips, with or without PLL, can be effectively used for the immobilisation of EVs in specific imaging techniques.

## 1 Introduction

Extracellular vesicles (EV) adhesive properties arise from phenomena including charge (the EV surface is usually negatively charged) and molecular affinities due to the presence of, for example, integrins and other adhesion molecules (Jimenez et al. 2019). Multiple studies have investigated EV interactions with specific materials, often in hopes of reducing negative consequences such as loss to surfaces during EV storage (Evtushenko et al. 2020; Ge et al. 2014; Görgens et al. 2022). However, direct immobilisation of EVs onto materials such as glass might also allow for the improvement of techniques, including fluorescence microscopy.

Traditionally, EVs have been captured for imaging using molecular affinity reagents such as antibodies (Reynolds et al. 2023; Singh et al. 2023). Alternatively, glass slides and coverslips can also be coated with poly‐L‐lysine (PLL) or poly‐D‐lysine (PDL). As polymerised amino acids with a cationic charge, these reagents enhance the capture of negatively charged particles (Yu et al. 2022; Altei et al. 2020).

Non‐specific immobilisation may be especially valuable now that super‐resolution microscopy (SRM) has evolved to enable semi‐quantitative multiplexed single‐molecule characterisation of the composition of single EVs and thus convey a richer sense of EV heterogeneity (Schürz et al. 2022). For this reason, we investigated several aspects of EV immobilisation onto surfaces for imaging. We tested the direct adherence of EVs onto borosilicate glass and quartz coverslips, further probing the extent to which pre‐coating with PLL enhances EV immobilisation. We also examined mounting conditions for SRM imaging and determined that glass‐immobilised EVs could also be used for cell interaction assays. We submit that these findings will be valuable for other investigators who wish to image immobilised EVs.

## 2 Materials and Methods

### 2.1 EV Production: Cell Culture and Transfection and EV Separation

Using the Expi293 expression system (Gibco #A14635), Expi293F cells were grown and transfected with the pLenti‐palmGRET reporter plasmid (Addgene #158221) (Wu et al. 2020) following the manufacturer's instructions, as described previously (Driedonks et al. 2022). Three days after transfection, the cell suspension was centrifuged at 300 × *g* for 5 min to remove cells from the conditioned medium. For Expi293F‐palmGRET EV separation, the conditioned medium was centrifuged at 2000 × *g* for 20 min, filtered through a 0.22 µm bottle‐top system (Corning) and concentrated 10X by tangential flow filtration (TFF, Vivaflow 50R TFF cassettes, Sartorius). Samples were further concentrated 4X using ultrafiltration (Centricon Plus 70 Ultracel PL‐100, Merck Millipore; 4000 × *g*, 20 min, RT) and eluted (1500 × *g*, 2 min). Samples were separated into 11 fractions using size‐exclusion chromatography (SEC; qEV10 70 nm columns, IZON). EV‐enriched fractions (1–4) were pooled and concentrated using Amicon 15 Ultra RC 10 kDa MWCO filters (Merck Millipore). Expi293F‐palmGRET EV aliquots in Dulbecco's phosphate‐buffered saline (DPBS) were stored at 4°C for short‐term use and at −80°C for long‐term storage.

### 2.2 Western Blotting

Expi293F‐palmGRET cells were harvested and washed twice in DPBS (2500 × *g*, 5 min, RT). The resulting pellet was lysed with RIPA buffer (Cell Signaling Technology #9806) for 1 h on ice, vortexing every 15 min. The cell lysate was centrifuged at 14,000 × *g* for 15 min at 4°C to remove debris, and the supernatant was quantified using BCA protein assay (Thermo Scientific #23227). EV samples and SEC fractions (10 µL) were lysed with RIPA buffer for 10 min at room temperature. Samples were mixed with reducing (Thermo Scientific #39000) or non‐reducing (Thermo Scientific #39001) sample buffer, boiled at 100°C for 5 min and loaded into Criterion TGX stain‐free gels (4%–15%; Bio‐Rad #5678084) with a Spectra Multicolor Broad Range Protein Ladder (Thermo Fisher #26634). Gels ran in Tris/Glycine/SDS buffer (Bio‐Rad #1610772) at constant voltage (100 V) for 1.5 h, and stain‐free gels were imaged on the Gel Doc EZ imager (Bio‐Rad). Proteins were transferred to PVDF membranes (iBlot 2 Transfer Stacks, Invitrogen #IB24001) on the iBlot 2 Gel Transfer Device (Invitrogen) using a stacked programme (20 V for 1 min; 23 V for 4 min and 25 V for 2 min). Membranes were blocked with 5% milk‐PBST for at least 1 h at room temperature under agitation and incubated with primary antibodies overnight at 4°C with orbital shaking. Membranes were probed for CD63 (1:3000, Ms, BD #556016), CD9 (1:3000, Ms, BioLegend #312102), syntenin (1:1000, Rb, Abcam #133267) and calnexin (1:1000, Rb, Abcam #22595), washed four times with 5% milk‐PBST for 5 min under agitation, and exposed to the appropriate secondary antibody (m‐IgGκ BP‐HRP, 1:5000, Santa Cruz #616102 or Mouse anti‐Rb IgG HRP, 1:5000, Santa Cruz #2327) for 1 h, RT under agitation. Membranes were washed four times with PBST, exposed for 60 s to SuperSignal West Pico ECL substrate (Thermo Scientific #34578), and documented using the iBright 1500 (Invitrogen).

### 2.3 Flow Nanoanalyzer

Expi293F‐palmGRET EV preparations were measured for particle concentration, size distribution and EGFP incidence (%) using a Flow NanoAnalyzer (NanoFCM Co., Ltd). System start‐up included laser alignment and calibration with fluorescent 250 nm silica nanoparticles (2.19E + 10 particles/mL, NanoFCM #QS2503) for particle concentration and a premixed silica nanosphere cocktail with monodisperse nanoparticle populations of 68, 91, 113 and 155 nm in diameter (NanoFCM #516M‐Exo) for size dispersion. Blank was set with DPBS as the vehicle. EV samples were diluted 1:1000 (v/v) in DPBS and boosted for 60 s before particle signal acquisition, using constant pressure of 1 kPa and event rates between 1500 and 10,000 events/min. Signals for side‐scattering and EGFP signal, particle concentration and size dispersion were calculated using NanoFCM Professional Suite V2.0 software. Experimental design and framework compliant with MIFlowCyt‐EV (Welsh et al. 2020) and MIFlowCyt (Lee et al. 2008) are detailed in the Supporting Information.

### 2.4 Transmission Electron Microscopy

Expi293F‐palmGRET EV stock was diluted 1:20 (v/v) and adsorbed to glow‐discharged carbon‐coated 400 mesh copper grids by flotation for 2 min. Grids were quickly blotted and rinsed by floatation on three drops of Tris‐buffered saline, 1 min each, before being negatively stained in two consecutive drops of 1% uranyl acetate (UAT) with tylose (1% UAT in deionised water (diH2O), double filtered through a 0.22‐µm filter). Grids were blotted and quickly aspirated to cover the sample with a thin layer of stain. Grids were imaged on a Hitachi 7600 transmission electron microscope (TEM) operating at 80 kV with an AMT XR80 CCD (8 megapixel), under magnification ranging from 30,000× to 120,000×.

### 2.5 Coverglass Chamber Preparation for EV Immobilisation

Schott D 263 coverglass chambers (μ‐Slide 8 Well high Glass Bottom, ibidi #80807) were coated with 300 µL of PLL solution (0.1% PLL in H_2_O (w/v), Sigma #P8920) and incubated overnight at 4°C. The coating residue was removed through two washes with 500 µL of DPBS (Gibco). Expi293F‐palmGRET EVs (4E + 08 particles/mL in DPBS, 300 µL per well) were used to coat both PLL‐coated and uncoated chambers and were incubated overnight at 4°C and protected from light. The next day, wells were washed with DPBS twice (300 µL per well) and filled with either 300 µL of DPBS or 100 µL of VECTASHIELD mounting medium (MM) (Vector Laboratories #H‐1000‐10). Samples were stored at 4°C until analysis. Three independent repeats with technical duplicates were done.

### 2.6 Coverslip Preparation for EV Immobilisation

Borosilicate glass coverslips (Carolina Cover Glasses, Circle 12 mm, Thickness 0.13–0.17 mm, Carolina Science & Math #NC9537307) and quartz coverslips (Ø10 mm × 0.25 mm thick, Ted Pella #26010) were rinsed with 70% ethanol and dried before use. Placed atop a 30 µL drop of EV dilution (4E + 08 particles/mL) on parafilm, coverslips were incubated overnight at 4°C, protected from light. Coverslips were rinsed twice with DPBS and placed onto clear histological slides using VECTASHIELD MM. Slides were sealed (CoverGrip Coverslip Sealant, Biotium #23005) and stored at 4°C until analysis. The experiment was conducted with two slips per group, in three independent repeats, using two distinct EV preparations.

### 2.7 Direct Stochastic Optical Reconstruction Microscopy (dSTORM)

A SRM system (Nanoimager, Oxford NanoImaging), coupled with NimOS software, was calibrated using beads detected using 405/473/532/635 nm laser excitation, mounted atop a 100× oil‐objective lens. Good quality channel mapping calibration was obtained through adequate point coverage. Coverslips and coverglass chambers were imaged in seven random sites (79.28 µm × 49.43 µm) with 1000 frames at 30 ms exposure and 1.4 numerical aperture. Laser intensity was optimised for each sample. Post‐acquisition spatial analysis was performed using CODI (alto.codi.bio), using the “clustering and counting” app for correcting drift, clustering populations and counting particles. Widefield images were acquired under 1× zoom, and reconstituted images were snapped acquired under 6–8× zoom.

### 2.8 B Cell Isolation

Non‐human primate (NHP) samples were obtained from SPF juvenile male pigtailed macaques *(Macaca nemestrina)* in procedures approved by the Johns Hopkins University Animal Care and Use Committee (ACUC # PR21M297) and conducted following the Weatherall Report, the Guide for the Care and Use of Laboratory Animals, and the USDA Animal Welfare Act. Blood samples were diluted (1:1, v/v) in Hank's balanced salt solution (HBSS) and applied to SepMate‐15 (IVD) tubes loaded with a density gradient (Percoll, Cytiva #17‐0891‐01). Blood was spun at 1200 × *g* for 10 min for separation into three phases, and the upper fraction, containing mononuclear cells (MNC), was transferred to fresh tubes and washed with HBSS. Cells were pelleted (300 × *g* for 8 min) and treated with red blood cell lysis buffer (ACK lysing buffer 0.83% NH_4_Cl, 0.1% KHCO_3_, 0.03% EDTA) for 10 min at 37°C. Cells were washed with HBSS, spun at 300 × *g* for 8 min and resuspended in cold selection buffer (PBS + 0.5% BSA + 2 mM EDTA) mixed with 20% (v/v) CD20 MicroBeads (Miltenyi Biotec #130‐091‐104 and #130‐042‐201) for 15 min at 4°C. The suspension was diluted in selection buffer, centrifuged at 300 × *g* for 8 min, resuspended in buffer, and applied onto MS columns (Miltenyi Biotec #130‐042‐201) for magnetic cell sorting using MiniMACS separators (Miltenyi Biotec). CD20^+^ B cells were collected in conical tubes, pelleted (300 × *g* for 8 min) and resuspended in RPMI 1640 + 10% FBS + 1% pen/strep for cell counting.

### 2.9 B Cell Adhesion to EV Coating

Coverglass chambers (μ‐Slide 8 Well High Glass Bottom, ibidi #80807) were coated with 300 µL PLL (0.1%, v/v) for 2 h at 4°C and washed with DPBS (300 µL/well). An EV suspension (4E + 08 particles/mL in DPBS) was used to coat wells overnight at 4°C, protected from light. Wells coated with 1% BSA‐PBS (200 µL/well) were used as the negative control. Excess coating was washed off with 300 µL DPBS, and non‐specific binding was blocked with 300 µL 1% BSA‐PBS in each well, followed by a 1‐h incubation at 37°C. B cells (1E + 05 cells in 300 µL RPMI 1640 10% FBS) were applied to each well and incubated at 37°C, 5% CO_2_, for 1 h. Unbound cells were removed with three washes with DPBS (300 µL per well) before fixation with 4% PFA for 10 min at RT. Cells were washed twice with PBS, permeabilised with 0.1% Triton X‐100 for 5 min at RT, and washed again. F‐actin was stained with Phalloidin‐647 (200 µL/well, 1:1000 dilution in PBS, Abcam #176759) for 1 h in the dark, and excess dye was washed off twice with PBS. Wells were coated with prolong MM with DAPI and sealed with parafilm for analysis using the Nanoimager (ONI). Cell count was assessed via nuclei count using FIJI (ImageJ), imaging a minimum of six random sites under 20× magnification using a confocal microscope (Zeiss 880 Airyscan FAST). The experiment was conducted on two independent occasions, using duplicate wells per group in each repeat.

### 2.10 Statistical Analysis

Raw data were checked for outliers using the ROUT method (Motulsky and Brown, 2006), assuming a *Q* = 0.1. D'Agostino‐Pearson omnibus K2 normality test was applied to identify data distribution. Parametric data were analysed using unpaired *t*‐test or one‐way ANOVA with Tukey's multiple comparison test, and non‐parametric data were analysed using the Mann‐Whitney test. Statistical analysis and graphs were executed on GraphPad Prism (v. 10.0.1 (170)).

## 3 Results

### 3.1 Expi293F‐palmGRET EV Characterisation

**FIGURE 1:**
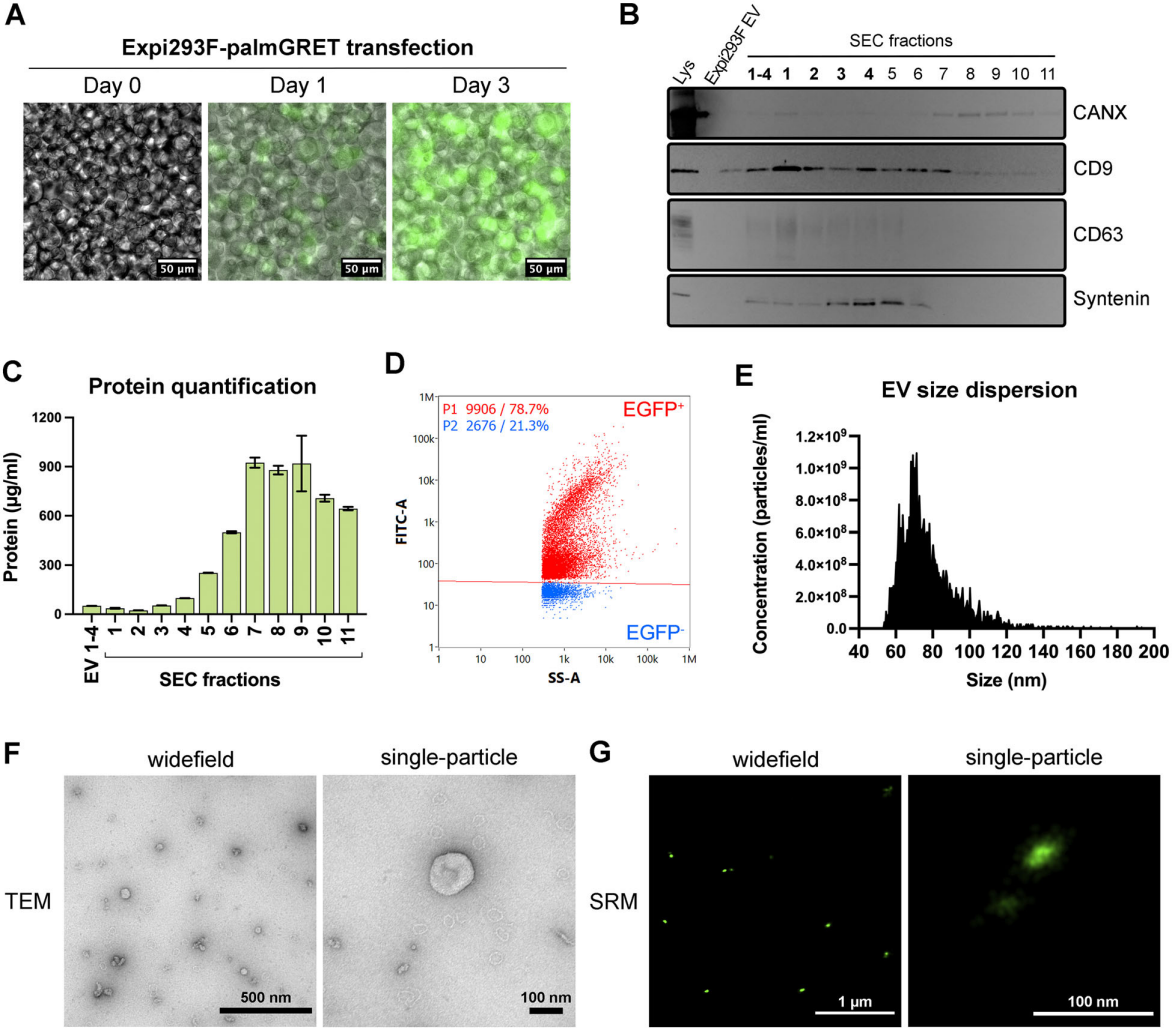
Expi293F‐palmGRET EV characterisation. (A) Fluorescence imaging of Expi293F cells tracking the 3 days of transient transfection with pLenti‐palmGRET. Scale bar: 50 µm. (B) Western blotting: membranes with Expi293F cell lysate and SEC fractions 1–11 were probed for calnexin (CANX), CD9, CD63, and Syntenin‐1. (C) Quantification of protein content in SEC fractions 1–11. (D) Scatter plot of EV nanoflow cytometry, showing 78.7% EGFP‐positivity amongst particles from the pooled SEC fractions 1–4. (E) Size distribution plot of Expi293F‐palmGRET EVs by nanoflow cytometry. (F) EV visualisation in widefield (left, scale bar: 500 nm) and zoomed‐in (right, scale bar: 100 nm) under transmission electron microscopy (TEM). (G) Super‐resolution imaging of EVs emitting EGFP fluorescence in widefield (left, scale bar: 1 µm) and zoomed‐in (right, scale bar: 100 nm).

Expi293F cells were cultured to 720 mL of culture. Approximately 2.8E + 9 cells were transfected with pLenti‐palmGRET EGFP‐nanoluciferase reporter plasmid. Transfection efficiency was estimated under a fluorescent microscope equipped with a 488 nm filter on the first and third days of transfection (Figure 1A). Cells reached a population of approximately 3.8E + 9 cells after 3 days, at which time EV‐containing conditioned culture medium was harvested and EVs were separated. Following the updated MISEV2023 guidelines (Welsh et al. 2024), Expi293F‐palmGRET EVs were characterised using multiple techniques. Volumetric Western blotting for proteins CD9, CD63, syntenin and calnexin indicated the expected depletion of ER‐specific calnexin and enrichment of EV‐related syntenin and tetraspanins CD63 and CD9 in the early SEC fractions (Figure 1B). Later SEC fractions were protein‐enriched compared with EV fractions (Figure 1C). EV‐enriched fractions (1–4) were pooled for nanoflow analysis, revealing concentration of 4.7E + 11 particles/mL, with 78.7% of particles being EGFP^+^ (Figure 1D). Additional information on the nanoflow analysis is available in the Supporting Information (Jimenez et al. 2019; Evtushenko et al. 2020). EV size dispersion peaked at 76 nm (Figure 1E). EVs were visualised with transmission electron microscopy (Figure 1F) and SRM (Figure 1G) at widefield and single‐particle levels, showing expected size, morphology and fluorescence.

### 3.2 EV Immobilisation Is More Efficient on Glass Than on Quartz Coverslips

**FIGURE 2:**
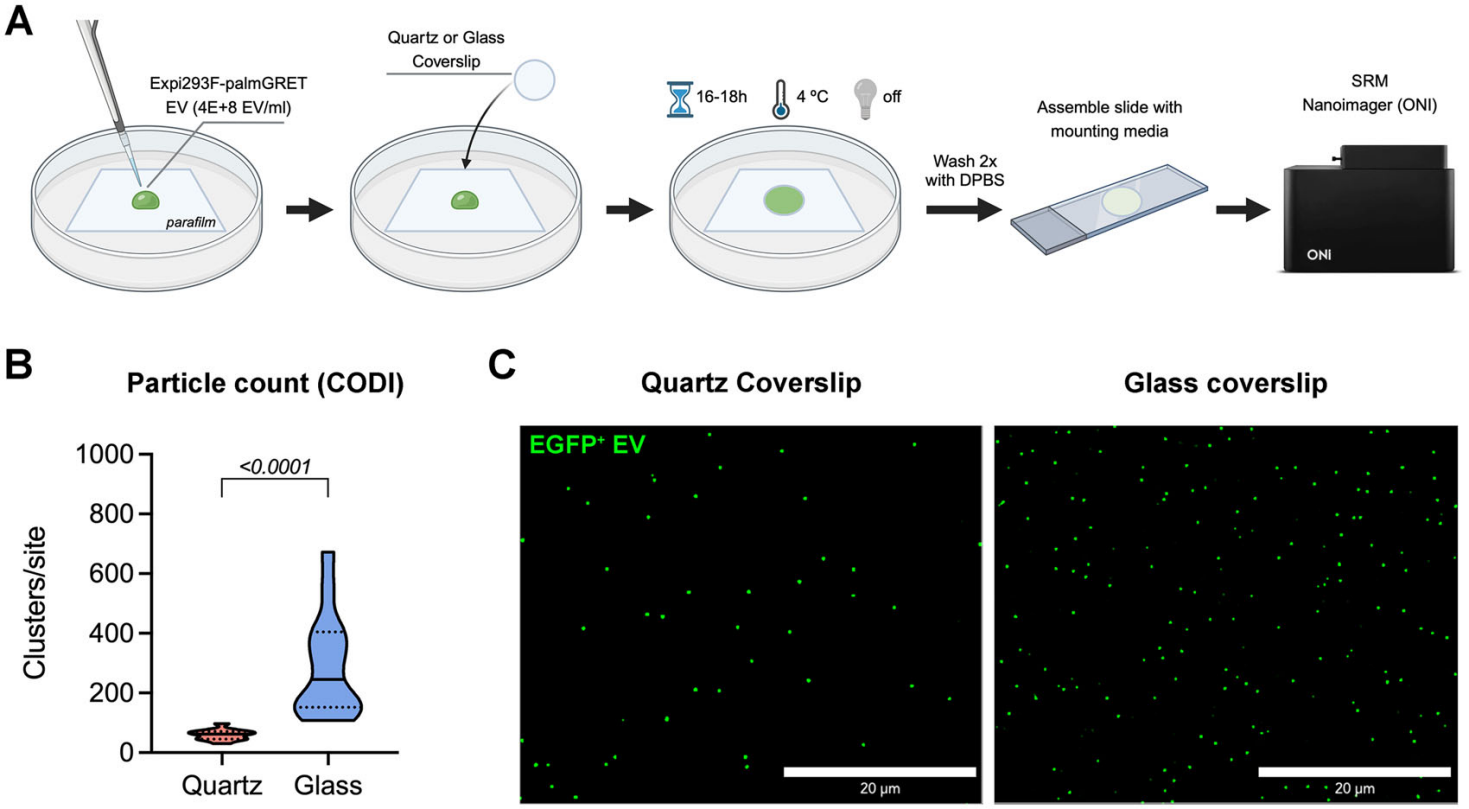
EV immobilisation on glass and quartz coverslips. (A) Experimental scheme for the preparation of EV‐coated coverslips for SRM imaging. (B) Comparison of particle count between quartz and glass coverslips, obtained using the CODI software (alto.codi.bio). Non‐parametric data was compared using the Mann‐Whitney unpaired *t*‐test, and the *p* value is displayed on the graph. (C) Representative images of rendered dSTORM frames, showing individual Expi293F‐palmGRET EVs in green, obtained at 60× magnification and 2× zoom. Scale bar: 20 µm.

Using the protocol depicted in Figure 2A, we found that both quartz and borosilicate glass can immobilise EVs. To count particles, each coverslip was imaged in at least six random sites in dSTORM, and the CODI software clustered signals from the multiple frames to identify individual EVs. Particle count detected 59.62 ± 17.67 on the quartz coverslip and 289.7 ± 172.3 clusters on the borosilicate glass coverslip, indicating a statistically significant difference in EV immobilisation (*p* < 0.0001) (Figure 2B). The inspection of the rendered images indicates the density of particles per frame (Figure 2C).

### 3.3 EV Immobilisation Is More Uniform With PLL Pre‐Coating

**FIGURE 3:**
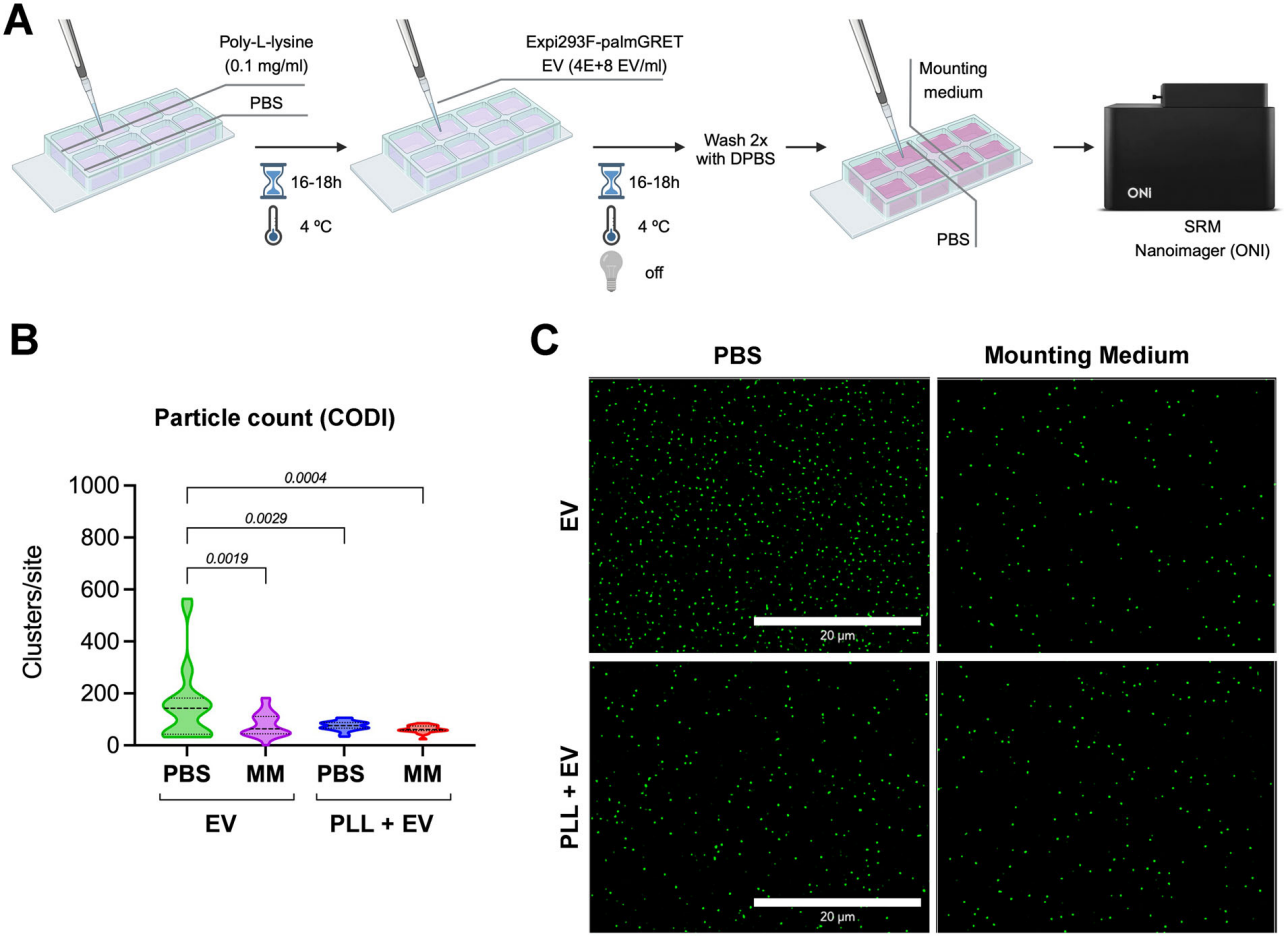
EV immobilisation onto borosilicate chamber coverglass. (A) Experimental scheme for the preparation of EV‐coated chamber coverglasses for SRM imaging. (B) Comparison of particle count between uncoated wells or pre‐coated with poly‐L‐lysine (0.1 mg/mL) prior to Expi293F‐palmGRET EV immobilisation and immersed in PBS or mounting medium (MM), obtained using the CODI software (alto.codi.bio). Non‐parametric data were compared using the Kruskal‐Wallis analysis of variance with Dunn's multiple comparison test, and the *p* values are displayed on the graph. (C) Representative images of rendered dSTORM frames, showing individual Expi293F‐palmGRET EVs in green, obtained at 60x magnification and 2× zoom. Scale bar: 20 µm.

Using an 8‐well culture chamber assembled atop a Schott D 263 borosilicate coverslip, we cross‐examined the requirement of specific MM and PLL to improve EV immobilisation on glass. The experimental design for this assay is shown in Figure 3A. Regarding the requirement of a MM to stabilise the sample for imaging in SRM, we found a statistically significant difference between PBS versus MM in glass surfaces only, but not in PLL‐coated glass (Figure 3B). This suggests that PBS may be a viable medium for immersing samples in culture chambers for dSTORM if used alongside PLL coating. We also found that EVs attached to borosilicate glass even without PLL coating (no PLL = 157.3 ± 144.9 clusters per site; PLL = 78.83 ± 45.26 clusters per site). Despite the no‐PLL condition showing the most EV clusters, it also showed a less uniform immobilisation, in which distinct sites had cluster values within a 532‐point range. As a comparison, the number of clusters per site in the PLL condition ranged with a 72‐point interval. Hence, the PLL condition may produce more consistent results. Reducing the number of procedural artifacts, optimising surface interaction and improving the consistency between experimental conditions. Representative images of the rendered frames indicating the density of particles per site are shown in Figure 3C.

### 3.4 B Cells Interact With PLL/Glass‐Immobilised EVs

**FIGURE 4:**
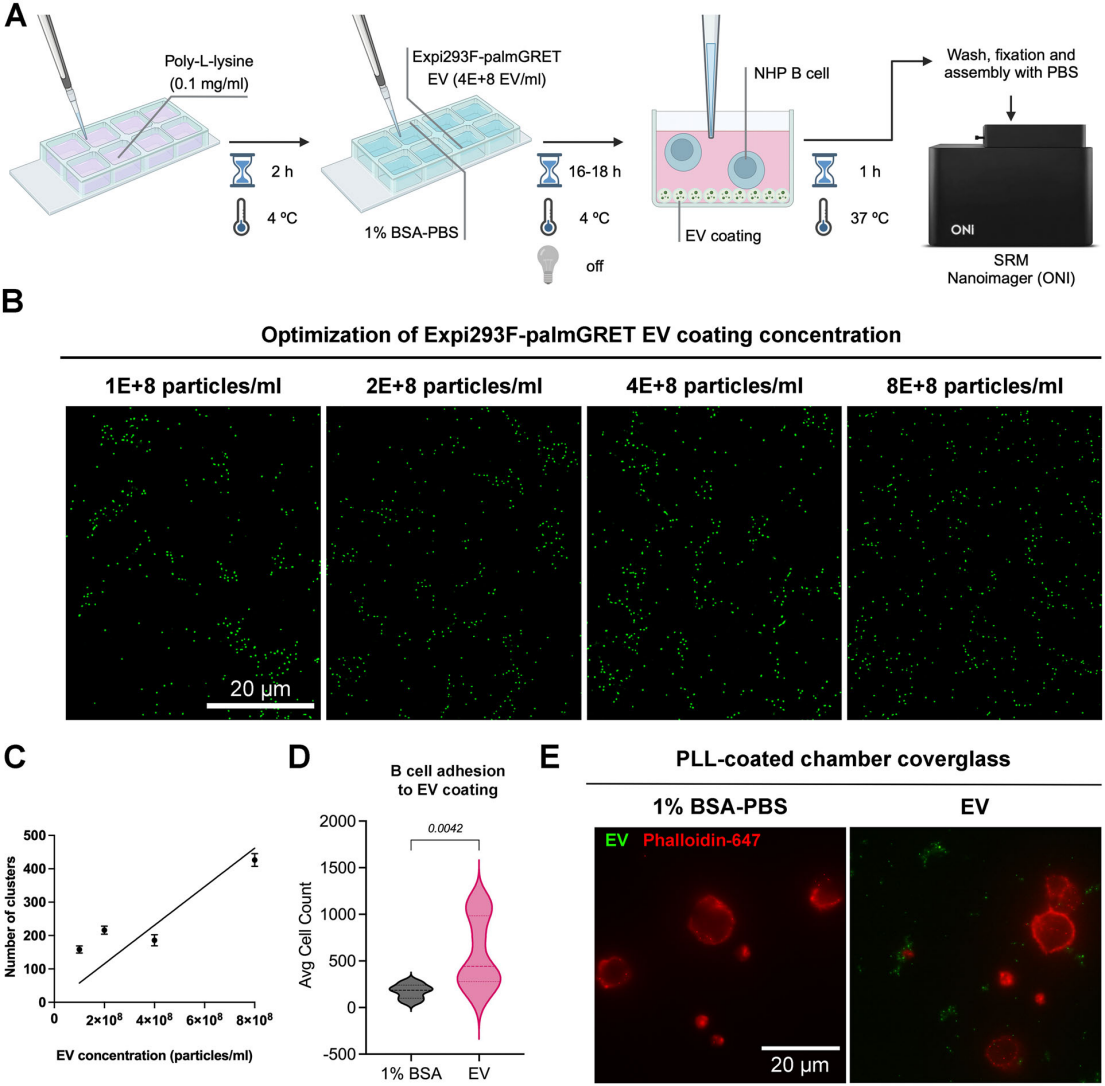
B cells adhere to EV coating. (A) Experimental scheme. (B) Optimisation of EV coating concentration, ranging from 1E + 8 to 8E + 8 particles/mL. Representative images from SRM rendering indicate Expi293F‐palmGRET EVs in green and were obtained at 60× magnification and 2× zoom. Scale bar: 20 µm. (C) Linearity of EV concentration per number of clusters detected using SRM. Cluster count was performed using CODI (alto.codi.bio). (D) Average count of attached cells in 1% BSA‐PBS (negative control) and EV coating after 1 h of incubation, obtained through nuclei counting. Non‐parametric data was compared using the Mann‐Whitney unpaired *t*‐test and the *p* value is displayed on the graph. (E) Representative SRM images from the adhesion assay, showing EVs (green) and NHP B cells (red). Scale bar: 20 µm.

We next performed a cell adhesion assay in which B‐cells were allowed to interact with an EV‐coated coverslip. This approach follows a previous report (Altei et al. 2020) and could be seen as an alternative to assays that interrogate the effects of cell orientation and local nanoparticle sedimentation on nanoparticle uptake (Cho et al. 2011). For this, we used PLL‐coated glass coverslips and NHP primary CD20^+^ B cells (Figure 4A). An optimisation step to adjust the EV concentration for this assay was performed, and an EV concentration of 4E + 8 particles/mL was chosen from a dilution series (Figure 4B) for subsequent comparison with a negative control (1% BSA‐PBS). Regression analysis determined a linear equation of *y* = 5.772E‐7*x, with a goodness of fit at 85.82% (Figure 4C). Although some cells bound to the negative control wells, on average, 3.3‐fold more cells adhered to EV‐coated wells (Figure 4D); the binding of cells was also confirmed by SRM (Figure 4E).

## 4 Discussion

Recently, a paper demonstrated that EV immobilisation occurs similarly in quartz glass slides and positively charged quartz slides whilst describing a novel plugin for particle count using standard fluorescent microscopy images (Schürz et al. 2022). Although optimising the conditions for an EV sedimentation assay (Cho et al. 2011), we pondered if similar results could be found between standard borosilicate glass coverslips and quartz coverslips, but instead using dSTORM to assess particle disposition. Using EGFP^+^ EVs, a significantly higher mobilisation of EVs in glass coverslips rather than quartz was observed, suggesting that the use of commercially available glass coverslips is possible for assays in which EVs need to be adsorbed by an amorphous surface. We also investigated if the use of PLL, a polycationic adhesive commonly used in microscopy preparations to prepare materials such as borosilicate glass for receiving biological samples (Taylor, 2008), could improve particle distribution throughout the surface. Our data suggest that the use of PLL coating in borosilicate glass is not required but aids the uniformity of EV distribution. We believe the PLL coating may contribute to EV binding in sites where the glass surface is not as homogeneous, by creating an electrostatic potential to improve EV adhesion (Schermelleh et al. 2019). On a side note, we also found that MM is not essential for SRM analysis, and PBS can be used as stable media to store microscopy samples under immersion for short periods.

Altogether, our data suggest that common lab materials, such as standard glass slides and saline buffer, can be used for certain applications, such as SRM, with adequate outcomes and reasonable quality. Although the use of specialised materials is often required for specific assays, including the use of imaging chambers (Xu et al. 2024) or EV profiling chips (Roseborough et al. 2023), their cost and availability may be limiting. Widely available, cost‐effective alternatives may broaden the reach of these technologies. However, we must emphasise that further optimisation may be required to understand how nanoparticle capture occurs for diverse particles, including EVs with various surface modifications as well as the very broad range of non‐vesicular extracellular particles (Simon et al. 2025; Pham et al. 2021). To the best of our knowledge, no studies so far have investigated whether PLL is required for EV immobilisation on glass surfaces, and few have studied the interaction of EVs with different materials for functional assays.

Sample preparation for specific applications such as total internal reflection fluorescence (TIRF) microscopy and dSTORM often require refined materials that improve their optical transmittance without compromising the roughness value on the surface or the chemical resistance (Schermelleh et al. 2019). Quartz is considered a superior material for optical transmission; however, its high softening point (1500°C) creates a more delicate material and a costlier product (Tang et al. 2021). A commercially available option is the standard glass coverslip, made from high‐quality borosilicate glass with different sizes and thicknesses (e.g., 0.09–0.25 mm), which are suitable for microscopy, especially when treated with PLL in preparation for cell adhesion (Mazia et al. 1975). EVs bind more efficiently to borosilicate glass than quartz, as found using dSTORM, suggesting that this material may be used for the development of new products focused on highly specific microscopy techniques.

Our findings suggest that the immobilisation of EVs for highly specific imaging techniques may be achieved by using standard lab materials such as borosilicate glass coverslips. This simpler approach to dSTORM broadens the possibilities for methods and applications that can employ SRM. Also, coupled with the similarity between PBS and MM to store the sample, we described a cheaper option for SRM preparation with equivalent results to costlier options.

## Author Contributions

**Bianca Pachane**: conceptualization (equal), data curation (lead), formal analysis (lead), investigation (lead), methodology (lead), project administration (equal), writing–original draft (lead). **Bess Carlson**: methodology (supporting), visualization (supporting). **Suzanne Queen**: methodology (supporting), visualization (supporting). **Heloisa Selistre‐de‐Araujo**: funding acquisition (supporting), supervision (equal), writing–review and editing (supporting). **Kenneth Witwer**: conceptualization (equal), funding acquisition (lead), resources (lead), software (lead), supervision (equal), writing–review and editing (lead).

## Conflicts of Interest

K.W.W. has been an advisory board member of ShiftBio, Exopharm, NeuroDex, NovaDip and ReNeuron; holds stock options with NeuroDex and privately consults as Kenneth Witwer Consulting. Ionis Pharmaceuticals, Yuvan Research and AgriSciX have sponsored or are sponsoring research in the Witwer laboratory. The other authors declare no conflicts of interest.

## Supporting information



Supporting Information

## Data Availability

We have submitted all relevant data of our experiments to the EV‐TRACK knowledge base (EV‐TRACK ID: EV240144) (Van Deun et al. 2017). The data that support the findings of this study are available from the corresponding author upon reasonable request.
