# Supplementary material for: Exploring the Adhesion Properties of Extracellular Vesicles for Functional Assays
Source: J Extracell Biol. 2025 Apr 25;4(4):e70042. doi: 10.1002/jex2.70042 (PMC12025881; doi:10.1002/jex2.70042)
Supplement: Supplementary file 1 — Supporting Information [file JEX2-4-e70042-s001.pdf]

Supplementary Information 1: MIFlowCyt-EV framework

Component	Description
**1.1**	EVs were separated from Expi293™ cells (Gibco #A14527) after three days of transfection with the pLenti-palmGRET reporter plasmid (Addgene #158221). EVs were collected by conditioned media centrifugation (300 x g for 5 min, 2,000 x g for 20 min), filtration (0.22 μm bottle-top system, Corning), tangential flow filtration (TFF, Vivaflow® 50R TFF cassettes, Sartorius), ultrafiltration (Centricon Plus 70 Ultracel® PL-100, Merck Millipore; 4,000 x g, 20 min, RT) and size-exclusion chromatography (SEC; qEV10 70nm columns, IZON). From the 11 fractions, EV-enriched fractions 1-4 were pooled and concentrated using Amicon 15 Ultra RC 10 kDa MWCO filters (Merck Millipore). Expi293F-palmGRET EV aliquots in Dulbecco's phosphate buffered saline (DPBS) were stored at 4 ºC for short-term use and at -80 ºC for long-term storage.
**1.2**	**1.1 Aim:** To characterize Expi293F-palmGRET EVs regarding their size, concentration and fluorescence following MISEV2023 guidelines. **1.2 Keywords:** EV; extracellular vesicles; characterization. **1.3 Experimental variables:** none.
**2.1**	The Expi293™ expression system (Gibco #A14635) was used to transiently transfect the pLenti-palmGRET reporter plasmid. Expi293 cells were seeded in sterile Erlenmeyer flasks in Expi293 growth media under a 3 x 10^6 cells/ml concentration and allowed to grow overnight at 37 ºC, 8% CO_2 under orbital shaker. Cells were diluted to a 3 x 10^6 cells/ml concentration in fresh media. Two solutions containing the ExpiFectamine™ 293 reagent or the pLenti-palmGRET plasmid (1 μg total plasmid DNA per mL of culture volume) in Opti-MEM™ I Medium were prepared and incubated for 5 minutes at room temperature. Both solutions were combined at room temperature for 10 min before being added to the cell culture while swirling. Cells were maintained at 37 ºC, 8% CO_2 for 18-22 hours, and checked for EGFP fluorescence using a microscope. A transfection enhancer was added to the cell culture, and the flasks were further incubated for two days. On day 3 after transfection, $EGFP^+$ EVs were collected from the conditioned media.
**2.2**	Cells were harvested from flasks and centrifuged at 300 x g for 5 min to separate from the conditioned media. The media was further centrifuged at 2,000 x g for 20 min to remove debris and filtered using a bottle-top system (0.22 μm). After tangential flow filtration and ultrafiltration, the conditioned media was separated using size-exclusion chromatography using DPBS as buffer.
**2.3**	1 μl of EV samples were diluted in 999 μl of DPBS for a 1:1000 dilution. For concentrated samples, a 100 μl aliquot of the 1:1000 dilution was further diluted in 900 μl of DPBS, resulting in a 1:10,000 dilution.
**3.1**	We used as buffer-only control a 0.22 μm filtered DPBS sample, which was recorded prior to the analysis using the same acquisition parameters. The buffer-only control had a count of ~0.98 events per second.
**3.2**	No buffer with reagents were used in this experiment.
**3.3**	No unstained controls were used in this experiment.
**3.4**	No isotope controls were used in this experiment.
**3.5**	No single-stained controls were used in this experiment.
**3.6**	No procedural controls were used in this experiment.
**3.7**	Samples were serially diluted for up to two times, with 1 μl of EV samples diluted in 999 μl of DPBS for a 1:1000 dilution. If samples were too concentrated, a 100 μl aliquot of this 1:1000 dilution was further diluted in 900 μl of DPBS, resulting in a 1:10,000

(continues next page)

Component	Description
**3.8**	No detergent-treated controls were used in this experiment.
**4.1**	Based on the buffer alone control (**Component 3.1**), detection was triggered on the 488 nm laser excited FITC channel (10-50 mW 488 laser) at a threshold of 13.7 arbitrary units, determined using quality control beads (0.25 µm Fluorescent Silica Microspheres) and the manufacturer's calibration values (see **Component 4.3** for fluorescence calibration). The buffer alone control had an event rate of ~1 events per second.
**4.2**	Samples were enumerated using the integral instrument flow rate sensors, resulting in a flow rate of 26.07 nL/min. This was calibrated using weighted volumes of deionized water prior to analysis.
**4.3**	Arbitrary FITC fluorescence scale units (channel number), excited by the 488 nm laser and collected using a 525/40 bandpass filter were converted to MESF units using 0.25 µm Fluorescent Silica Microspheres (250nm Std Fi SiNPs, NanoFCM Inc).
**4.4**	Side scatter calibration was performed on the NanoAnalyzer software (NanoFCM Inc), considering the wavelength (405 nm) and polarization state (perpendicular to detection of the laser), the light collection geometry (side scatter, numerical aperture) and the particle diameter and refractive index. Side scatter decay was reported at 10%.
**5.1**	EV diameter was approximated using a calibration curve determined by silica nanospheres size and side scattering intensity. The silica nanosphere cocktail contained particles with diameters ranging from 68~155 nm (S16M-Exo, NanoFCM Inc), peaking at 68, 91, 113 and 155 nm. Beads were boosted under 10 mW laser power, 10% SS decay and ≤1.0 kPa sampling pressure.
**5.2**	Particle refractive index was derived from the ratio of side and forward scatter signal following the silica nanosphere calibration curve described in **Component 5.1**.
**5.3**	No EV epitopes were evaluated in this experiment.
**6.1**	The MIFlowCyt checklist v.1.0.0 has been completed and attached in the Supplementary Information
**6.2**	The calibrated FITC channel detected nanoparticles ranging from 40-200 nm diameters and up to 407,000 FITC MESF units. Positive events on FITC were assumed to be higher than a threshold of 57.8 FITC MESF units.
**6.3**	The detected concentration of Expi293F-palmGRET EVs between calibrated detection range (57.8 and 407,000 FITC MESF units) was 4.65 x 10^{11} particles per ml. Flow cytometer acquisition settings were maintained for all samples, including triggering threshold, voltages and flow rate.
**6.4**	EV brightness was determined as a 78.7% fraction of the total event count, corresponding 9906 of the 12582 events recorded. EGFP brightness in EV samples was not quantified.
**7.1**	NFA files and reports are available upon reasonable request by contacting the corresponding author.

Supplementary Information 2: MIFlowCyt-compliant experimental design

Component	Description
**1. Experimental Overview**	
**1.1 Purpose**	To characterize Expi293F-palmGRET EVs regarding their size, concentration and fluorescence following MISEV2023 guidelines
**1.2 Keywords**	EV; extracellular vesicles; characterization.
**1.3 Experimental Variables**	None.
**1.4 Organization**	***1.4.1 Name****:* Johns Hopkins University School of Medicine<br>***1.4.2 Address:*** 733 N Broadway, Baltimore, MD 21205, United States
**1.5 Primary Contact**	***1.5.1 Name:*** Dr. Kenneth Whitaker Witwer<br>***1.5.2 Email Address:*** kwitwer1@jhmi.edu
**1.6 Date**	Flow cytometry analysis was performed on Feb 2, 2023.
**1.7 Conclusions**	We obtained an EV preparation with a concentration of 4.65 x 10^{11} particles per ml and 78.7% of particles were positive for EGFP fluorescence.
**1.8 Quality Control Measures**	The system was calibrated using quality control beads and a vehicle control (i.e., DPBS) was used as a blank.
**2. Flow Sample/Specimen Details**	
**2.1 Sample/Specimen Material Description**	
2.1.1 Biological Samples	*2.1.1.1 Biological Sample Description:* EVs separated from the conditioned media from Expi293F human cells (Gibco # A14527) transfected using the Expi293F™ expression system (Gibco # A14635) with the plasmid pLenti-palmGRET (Addgene #158221).<br>*2.1.1.2 Biological Sample Source Description:* The Expi293F cells are derived from the HEK 293 human embryonic kidney cell line and are grown under suspension in Expi293™ Expression Medium (Gibco #A1435101).<br>*2.1.1.3 Biological Sample Source Organism Description:* EVs separated from the conditioned media from Expi293F human cells transfected using the Expi293F™ expression system with the plasmid pLenti-palmGRET.<br>2.1.1.3.1 Taxonomy: *Homo sapiens*<br>2.1.1.3.4: Phenotype: Expi293F human cells are derived from the 293 cell line, and are a core component of the Expi293 Expression System. They are maintained in suspension culture and will grow to high density in Expi293 Expression Medium. Expi293F cells are highly transfectable and generate superior protein yields compared to standard 293 cell lines in transient protein expression.<br>2.1.1.3.5: Genotype: Undisclosed by the manufacturer (Thermo Fisher Scientific).

(continues next page)

Component	Description
	2.1.1.3.6 Treatment: The Expi293™ expression system (Gibco #A14635) was used to transiently transfect the pLenti-palmGRET reporter plasmid. Expi293 cells were seeded in sterile Erlenmeyer flasks in Expi293 growth media under a 3 x 10^6 cells/ml concentration and allowed to grow overnight at 37 ºC, 8% CO_2 under orbital shaker. Cells were diluted to a 3 x 10^6 cells/ml concentration in fresh media. Two solutions containing the ExpiFectamine™ 293 reagent or the pLenti-palmGRET plasmid (1 μg total plasmid DNA per mL of culture volume) in Opti-MEM™ I Medium were prepared and incubated for 5 minutes at room temperature. Both solutions were combined at room temperature for 10 min before being added to the cell culture while swirling. Cells were maintained at 37 ºC, 8% CO_2 for 18-22 hours, and checked for EGFP fluorescence using a microscope. A transfection enhancer was added to the cell culture, and the flasks were further incubated for two days. On day 3 after transfection, $EGFP^+$ EVs were collected from the conditioned media.
**2.3 Sample Treatment(s) Description**	None
**2.4 Fluorescence Reagent(s) Description**	EV samples were separated after transfection with the EGFP tag and detected accordingly to the following optical detectors:<br>- *Reporter for FL1:* SS<br>- *Reporter for FL2:* FITC<br>- *Reporter for FL3:* PC5
**3 Instrument Details**	
**3.1 Instrument Manufacturer**	NanoFCM Inc<br>https://www.nanofcm.com
**3.2 Instrument Model**	Flow NanoAnalyzer<br>https://www.nanofcm.com/category/productorcenter/flownanoanalyzer/
**3.3 Instrument Configuration and Settings**	Instrument configuration and settings were consistent with the original installation standard (June 28, 2018).

Supplementary Information 3: Nano-Flow Cytometry Report

Size & Concentration Report

1xSEC fraction 1

Data File	20230202 1xSEC fraction 1 5.nfa	Population	Total

SN: FNAN30E20112669
Software: V2.0
Sample Pressure: 1.0Kpa

Laser: 10/50 mW 488
SS Decay: 10%
Threshold/sub: 95.3 13.7 86.5 1/0 0 0 0
Min Width: 0.3 ms

Total Size Infomation

All Events	12116	
Gating Events	12116	
% of all	100.00	
Median	72.75	nm
Mean	76.02	nm
Std Dev.	13.27	nm

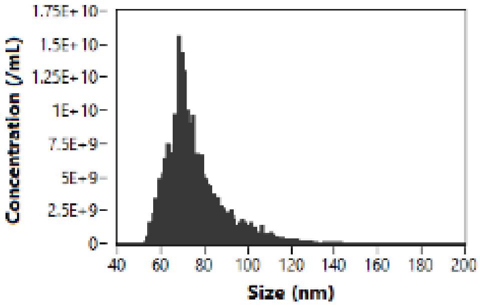

Total Concentration Infomation

	Particle Number	Dilution Factor
STD	5709	100
Blank	466	——
Sample	12582	1000

STD Con.	2.19E+10	Particles/mL
Sample Flow Rate	26.07	nL/min
Sample Con.	4.65E+11	Particles/mL

Corrected Ratio: 12116/12116 100.0%

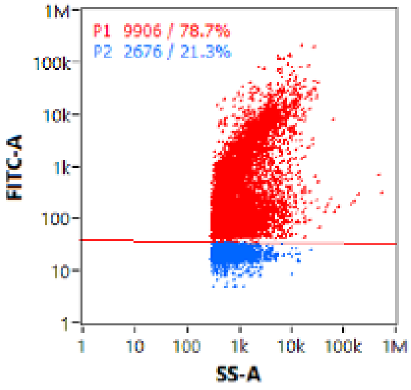

Report By :

2/2/2023 1:54 PM
